# Overcoming limitations of self-report: an assessment of fear of weight gain in anorexia nervosa and healthy controls using implicit association tests

**DOI:** 10.1186/s40337-021-00379-8

**Published:** 2021-02-18

**Authors:** Tiana Borgers, Nathalie Krüger, Silja Vocks, Jennifer J. Thomas, Franziska Plessow, Andrea S. Hartmann

**Affiliations:** 1 Department of Psychiatry and Psychotherapy, University of Münster, Münster, Germany; 2 Unit of Clinical Psychology and Psychotherapy, Institute of Psychology, Osnabrück University, Knollstr. 15, 49069 Osnabrück, Germany; 3 Eating Disorders Clinical and Research Program, Department of Psychiatry, Massachusetts General Hospital and Harvard Medical School, Boston, MA USA; 4 Neuroendocrine Unit, Department of Medicine, Massachusetts General Hospital and Harvard Medical School, Boston, MA USA

**Keywords:** Anorexia nervosa, Implicit association test, IAT, Drive for thinness, Fat phobia, Implicit association, Fear of weight gain, Feeding and eating disorders

## Abstract

**Background:**

Fear of weight gain is a characteristic feature of anorexia nervosa (AN), and reducing this fear is often a main target of treatment. However, research shows that 20% of individuals with AN do not report fear of weight gain. Studies are needed that evaluate the centrality of fear of weight gain for AN with a method less susceptible to deception than self-report.

**Methods:**

We approximated implicit fear of weight gain by measuring implicit drive for thinness using implicit association tests (IATs). We asked 64 participants (35 AN, 29 healthy controls [HCs]) to categorize statements as *pro-dieting* vs. *non-dieting* and *true* vs. *false* in a questionnaire-based IAT, and pictures of *underweight* vs. *normal-weight* models and *positive* vs. *negative* words in a picture-based IAT using two response keys. We tested for associations between implicit drive for thinness and explicitly reported psychopathology within AN as well as group differences between AN and HC groups.

**Results:**

Correlation analyses within the AN group showed that higher implicit drive for thinness was associated with more pronounced eating disorder-specific psychopathology. Furthermore, the AN group showed a stronger implicit drive for thinness than HCs in both IATs.

**Conclusion:**

The results highlight the relevance of considering fear of weight gain as a continuous construct. Our implicit assessment captures various degrees of fear of weight gain in AN, which might allow for more individually tailored interventions in the future.

## Plain English summary

While fear of weight gain is the main known reason for food intake restriction in individuals with anorexia nervosa, some individuals deny experiencing it. It is relevant to know whether fear of weight gain a) actually does not exist in these individuals, b) cannot be consciously perceived, or c) is denied, as this might impact choice of intervention strategies and reduce treatment drop-out found in this group. As self-reports might be biased, other assessment methods less susceptible to deception need to be implemented. Since the reasons for food intake restriction vary across and within individuals with anorexia nervosa, fear of weight gain should be viewed as a dimensional variable in this context. Therefore, the aim of our study was to examine associations between implicit fear of weight gain and other eating disorder symptoms. We assessed fear of weight gain by measuring drive for thinness, with the latter not being an identical but closely related construct that has shown to differentiate between individuals with anorexia nervosa reporting vs. denying fear of weight gain in explicit measures. Therefore, we used two implicit tasks in a sample of individuals with anorexia nervosa and healthy controls. Results indicate that implicit drive for thinness is associated with a range of eating disorder-specific and general psychopathological symptoms. In addition, individuals with anorexia nervosa showed greater implicit drive for thinness than healthy controls. These results illustrate the importance of assessing implicit drive for thinness in individuals with anorexia nervosa for an ultimate adaptation of therapeutic interventions according to the individual level of fear of weight gain, both explicitly reported and implicitly measured.

## Background

Anorexia nervosa (AN) is a severe and chronic mental disorder with one of the highest mortality rates among all mental illnesses [1]. The efficacy of psychological interventions is limited [2, 3] and needs to be increased through an updated understanding of the central and potentially maintaining features of this disorder [4]. Body image disturbance—one of the key symptoms—is characterized by a multifactorial pattern of inappropriate attitudes and emotions toward the body, such as body dissatisfaction or fear of weight gain [5]. While body dissatisfaction in AN has been intensively studied [6], research on fear of weight gain (fat phobia, FP) is still lacking. Cognitive-behavioral theories have acknowledged that FP is particularly relevant for body self-schema activation, leading to disorder-specific cognitive biases and behaviors, with resulting distortions leading to a reinforcement of this fear [7]. Hence, early research stated that FP is central to the phenomenology of AN, and it was assumed to be the main reason for food intake restriction and a central diagnostic criterion in the *Diagnostic and Statistical Manual of Mental Disorders fourth edition* (DSM-IV-TR) [8]. In the DSM-5, however, an explicit expression of FP is no longer a sine qua non criterion of AN, as long as persistent behavior that counteracts weight gain is demonstrated [9]. Indeed, research suggests that 15–20% of individuals with AN do not report FP [10, 11]. These individuals with non-fat-phobic AN (NFP-AN) have shown lower scores on the restraint, eating concern, weight concern, and shape concern subscales of the Eating Disorder Examination (EDE [12]) and on the body dissatisfaction subscale of the Eating Disorder Inventory – 2 (EDI-2 [13]) than individuals with fat-phobic AN (FP-AN [10, 14, 15]). Additionally, they have exhibited fewer bulimic symptoms [14]. With regard to general psychopathology, individuals with NFP-AN typically present with lower depression and trait anxiety compared to individuals with FP-AN [11, 15, 16]. Furthermore, individuals with NFP-AN show significantly higher drop-out rates [11], higher standardized mortality rates [17], and reduced insight into their condition [11]. Accordingly, differences between individuals with FP-AN and NFP-AN may also be due to a lack of conscious awareness of the underlying FP [18]. Other reasons for differences in reported FP might be due to difficulties in identifying and describing their emotions (i.e., alexithymia [19]) or a tendency to deny both their low weight and AN [20] and potentially also FP in avoidance of treatment. Thus, a closer assessment of FP is essential in order to either modify treatment strategies depending on individual levels of FP or to resolve underlying shape and weight concerns in individuals denying or lacking awareness of FP, increasing efficacy of psychological interventions.

The aforementioned studies have mainly assessed FP by self-report [21, 22], which is problematic due to its susceptibility to intentional and unintentional deceptions as outlined above [18]. Implicit measurement methods might provide a solution for a more reliable assessment of FP [23]. One of the most common instruments is the Implicit Association Test (IAT [24]). The original IAT asks individuals to categorize stimuli into four categories and category-combinations. The faster the categorization of stimuli to one of the four categories (two opposing ones on each side), the stronger the implicit association of the two constructs on one side [25]. In clinical research, disorder-specific implicit attitudes have been successfully assessed, for instance, in social anxiety disorder [26], specific phobias [27], and obsessive-compulsive disorder [28].

In eating disorders (EDs), the IAT has been successfully employed to measure implicit ED-related attitudes [29–32]. To date, only one study has used the IAT to assess drive for thinness in individuals with FP-AN and NFP-AN, avoidant/restrictive food intake disorder (ARFID), and healthy controls (HCs [33]). Izquierdo et al. [33] employed a questionnaire-based IAT (qIAT [34]) to assess the association between the categories *pro-dieting/non-dieting* and *true/false* statements, and a picture-based IAT (pIAT) to assess the association between the categories *underweight/normal-weight* models and *positive/negative* words. Drive for thinness is not identical to FP but a closely related construct that has been shown to differentiate between individuals with AN reporting vs. denying FP in explicit measures of drive for thinness [10, 11, 35]. Therefore, Izquierdo et al. [33] examined implicit drive for thinness as an approximation for implicit FP using two IATs. The results revealed stronger associations between *pro-dieting* and *true* statements in participants with both FP-AN and NFP-AN. In contrast, a stronger association between *pro-dieting* and *false* statements was observed in HCs. In the pIAT, HCs demonstrated a stronger association between *underweight* models and *negative* words than did individuals with FP-AN and NFP-AN. While individuals with FP-AN and NFP-AN differed in their self-reported FP, groups did not differ in their implicit association between *pro-dieting* and *true* statements in the qIAT. Additionally, both groups showed a similar difference from HCs in the pIAT, highlighting comparable implicit biases. However, the study did not take a dimensional approach, as it did not examine the association between implicit drive for thinness and psychopathology.

As illustrated above, studies investigating AN and FP are mainly based on a categorization into FP-AN vs. NFP-AN. However, taxometric studies [36, 37] have pointed mainly to a dimensional interpretation of ED pathology. Since ED pathology in general has shown to present dimensionally [36], and specific aspects, such as delusionality of appearance-related beliefs, vary widely across individuals with AN [38], dimensional models may contribute to a better understanding of the heterogeneity of AN, resulting in stronger implications for considering FP as a continuous variable [36, 37]. This in turn would allow for a promotion of specific dimensional assessments and individually adapted treatments of individuals with AN on a continuum of FP. Therefore, the current study aimed to test whether implicitly measured drive for thinness, as a differentiating variable for FP, is associated with explicitly reported ED-specific and general psychopathology within AN, complementing the categorial findings of Izquierdo et al. [33] with a novel dimensional approach. As there is no comparable research approach on implicit drive for thinness in individuals with AN from a different culture than the United States (US) - American cultural background, the present study also aimed to extend these previous findings [33] to a German sample. Participants underwent a pIAT as well as a qIAT (see [33]), accordingly including pictures of *underweight* and *normal-weight* models as well as *pro-dieting* and *non-dieting* statements. A stronger bias toward *underweight* models or *pro-dieting* statements would represent implicit drive for thinness, while a stronger bias toward *normal-weight* models and *non-dieting* statements would be interpreted as healthy. Additionally, the participants completed a set of self-report measures regarding ED-specific and general psychopathology. We hypothesized that (1) stronger implicit drive for thinness would be associated with higher scores on ED-specific and general psychopathology in the AN group and that (2) participants with AN, but not HCs, would exhibit an implicit drive for thinness in the IATs.

## Methods

### Participants and recruitment

Participants were recruited upon admission to outpatient and residential facilities, via advertisements in newspapers and on thematic websites, from the student population, and postings in therapeutic and counseling institutions. Inclusion criteria were female gender, age ≥ 15 years, sufficient knowledge of the German language, and absence of neurological diseases (relevant for other parts of the project). The inclusion criteria for the AN group were a score of ≥2 on the SCOFF questionnaire [39], a screening instrument for identifying EDs, followed by a diagnosis of AN in the Structured Clinical Interview for DSM-IV (SCID; German version [40]). This DSM-IV SCID version [40] was applied, as the German version of the DSM-5 SCID [41] was first released in 2019. In order to comply with DSM-5 criteria of AN, we omitted the amenorrhea criterion and allowed a diagnosis of AN in the absence of FP if persistent behaviours counteracting weight gain were present in the setting of low weight and lack of recognition of associated medical consequences. Exclusion criteria for the AN group were a current psychotic and/ or manic episode, substance misuse or related addiction, and a body mass index (BMI) of 18.5 to 25 kg/m^2^. For HCs, exclusion criteria were any current or lifetime mental disorder and a BMI below 18.5 kg/m^2^. After the screening of *N* = 118 interested participants (HC: *n* = 62; AN: *n* = 56), *n* = 51 were excluded due to not meeting inclusion criteria (in HC: age or BMI too low, or presence of mental disorder; in AN: age too low or BMI too high). After diagnostic assessment, another participant needed to be excluded for not meeting diagnostic criteria for AN. A total of *N* = 66 participants took part in the study (HC: *n* = 29; AN: *n* = 37). Two participants with AN had to be excluded after participation in the study due to missing data, resulting in *N* = 64 participants (HC: *n* = 29; AN: *n* = 35). A power analysis indicated that we had the power to detect medium to large effect sizes (*f* = 0.38) in the main analyses between participants with AN and HCs.

### Procedure

First, a telephone prescreening was completed. Participants provided written informed consent (along with the parents if participants were minors) and underwent the following two diagnostic clinical interviews conducted by the senior author: The SCID for DSM-IV [40] and the EDE [12]. The IATs were conducted as part of a larger research project, which involved the following series of assessments: First, a picture was taken of each participant, which was used within the experimental tasks following the IAT paradigms, and a conjoint analysis was conducted. This paradigm has been described elsewhere [42]. The two IAT paradigms followed. Then eye-tracking [43], encephalography, and electromyography paradigms were conducted (the two latter yielded still unpublished data). Finally, participants completed a questionnaire battery (see Questionnaires). At the end of the study, participants were informed about the objectives of the study and received a reimbursement of 70 Euros. The study procedure was approved by the University’s ethics committee.

### Implicit association tests

The two tasks were identical to those used by Izquierdo et al. [33], with the contents professionally translated into German. They were programmed and presented using Presentation 20.1 (Neurobehavioral Systems Inc., Berkeley, CA, USA) on a Dell Latitude E5520 laptop (2.40GHz, 4.00GB) on a 16.5 in. HD WLED screen (resolution 1920 × 1080, color depth 32 bit). In both tasks, items were presented in random order in the middle of the screen, and participants were asked to categorize them into categories or category-combinations, indicated in the upper corners of the screen, via keystrokes. In two blocks, two sets of categories were displayed in the upper corners. The strength of association between two combined categories within an individual was operationalized as the speed with which the individual categorized items accordingly. The stronger the association, the easier and faster the categorization [32]. For both IATs, we followed the established block structure of Greenwald, Nosek, and Mahzarin [44]. To avoid sequence effects [45], two randomly assigned pIAT and qIAT versions were created, with different sequences of blocks (59.4% of participants completed version 1 and 40.6% version 2).

#### Picture-based implicit association test (pIAT)

Categories presented in the pIAT included *underweight* models or *normal-weight* models and *positive* words or *negative* words [32, 33]. Presented stimuli comprised five pictures of underweight vs. normal-weight models, and five words with a positive valence (e.g., “happy”) vs. words with a negative valence (e.g. “awful”). For example, a faster categorization of a picture of an underweight model into the category *underweight* when combined with the category *positive* words (as compared to the opposite pairing) would be interpreted as implicit FP. Comparable with psychometrics of previous studies [44], the pIAT obtained an internal consistency of *r* = .62 (*p* < .001).

#### Questionnaire-based implicit association test (qIAT)

The qIAT used in this study is conceptually based on the qIAT by Yovel and Friedman [34] and Izquierdo et al. [33]. The four categories *pro-dieting* vs. *non-dieting* statements and *true* vs. *false* statements each comprised five statements that could be categorized accordingly, e.g., “I am terrified of gaining weight” vs. “My weight rarely enters my mind” and “I’m participating in an experiment” vs. “I’m climbing a steep mountain”. In this case, a faster categorization of a *pro-dieting* statement into the *pro-dieting* category, when combined with the category *true* statements, would be interpreted as implicit FP. In this study, the qIAT demonstrated an internal consistency of *r* = .70 (*p* < .001), which is in line with previous studies [34].

### Questionnaires

#### The SCOFF questionnaire (SCOFF [39])

The SCOFF is a screening tool for EDs and was employed in the current sample to detect potential participants with psychopathological tendencies toward AN. It contains five questions addressing core features of AN and bulimia nervosa. In this study, we used German translations previously validated by Berger et al. [46] and Richter et al. [47]. The internal consistencies in German samples varied between *α* = .45 and *α* = .66 [46, 47].

#### Structured Clinical Interview for DSM-IV (SCID; German-language version [40])

The SCID is a structured clinical interview used to diagnose DSM-IV Axis I disorders. In the current sample it was used for the diagnosis of AN and comorbid mental disorders. The interrater reliabilities were satisfactory to good (0.61 ≤ *r*_icc_ ≤ 0.83).

#### *Eating Disorder Inventory* – (EDI-2; German-language version [13])

The EDI-2 is a questionnaire assessing ED pathology. It consists of 11 subscales, of which the following were considered relevant as they are closest to fat-phobic psychopathology and used for further analyses in this study: body dissatisfaction, bulimia and drive for thinness. Items are rated on a six-point Likert scale from never (1) to always (6), and sum values were calculated as subscale scores. Internal consistencies of the EDI-2 subscales used in the present study were good to excellent (.73 ≤ *α* ≤ .94).

#### *Eating Disorder Examination* (EDE; German-language version 17.0D [12])

As a structured interview, the EDE assesses specific ED pathology on four subscales: restraint, eating concern, weight concern, and shape concern. All four subscales were considered relevant and used for further analyses in this study. Items are rated on a seven-point Likert scale from feature is not present (0) to feature is present every day/ to an extreme degree (6), and mean values were calculated as subscale scores. In the current sample, internal consistencies for the subscales were good to excellent (.70 ≤ *α* ≤ .95).

#### *Beck Depression Inventory-II* (BDI-II; German-language version [48])

This questionnaire assesses the level of depressive psychopathology. It consists of 21 items, each containing four statements varying in intensity or frequency, regarding the last two weeks. A total sum score was calculated for further analyses. In this study, the total score demonstrated an excellent internal consistency (*α* = .94).

#### *State-Trait Anxiety Inventory* (STAI; German-language version [49])

The STAI serves to capture fear as a state (state anxiety) and fear as a trait (trait anxiety). The trait version of the scale (Trait Anxiety scale, STAI-T) contains 20 items, which are based on statements regarding how the participants generally feel on four-point rating scales estimating frequencies from almost never (1) to almost always (4). A total sum score was calculated for the analyses. In this study, the internal consistency of the STAI-T was excellent (*α* = .96).

### Data analysis

Data preparation and statistical analyses were conducted in Microsoft Excel (Microsoft, Redmond, USA) and SPSS 25 (IBM, Armonk, USA). To compare demographic characteristics, ED-specific psychopathology and general psychopathology between groups, we conducted two-sample *t*-tests and Mann-Whitney *U*-tests.

Data preparation of IAT data was based on the improved algorithm criteria by Greenwald et al. [44] and included adjustments depending on reaction times and incorrect responses, as well as calculation of the test values, the D-scores. Mean D-scores for each group in each IAT provided information about the direction as well as the strength of the association. The D-scores in this study are positive if the associations between *pro-dieting* statements and *true* statements or *underweight* models and *positive* words are stronger than the associations between *pro-dieting* statements and *false* statements or *underweight* models and *negative* words. The D-scores can be interpreted equivalently to Cohen’s *d* effect sizes (small: *d* = 0.2; medium: *d* = 0.5; large: *d* = 0.8 [50]). In this study, the internal consistency of each IAT was calculated as the correlation between a D-score based on the practice blocks (Blocks 3 and 6) and a D-score based on the critical blocks (Blocks 4 and 7; see [44]).

To test our hypothesis that IAT D-scores would be positively correlated with ED-specific and general psychopathology in the AN group, we calculated Pearson correlation coefficients across all participants with AN to analyze interrelations of IAT D-scores with BDI-II and STAI-T scores. Relationships with non-parametric variables, such as BMI, EDE subscale scores (restraint, eating concern, weight concern, and shape concern), and EDI-2 subscale scores (body dissatisfaction, bulimia, and drive for thinness), were calculated using Spearman correlation coefficients. Furthermore, to test our hypothesis that participants with AN would exhibit significantly higher pIAT and qIAT D-scores than the HC group, we conducted two-sample *t*-tests.

## Results

### Sample characteristics

Fourteen participants with AN (40%) had at least one comorbid disorder, with the major depressive disorder being the most common (17.14%). As expected, participants with AN scored significantly higher on explicit measures of ED-specific and general psychopathology. As per study design, average BMI was significantly lower in the AN group than in HCs. Detailed sample characteristics are depicted in Table 1.

**Table 1:** Differences in demographic, anthropometric, and clinical variables between AN and HC

			AN *n* = 35	HC *n* = 29	Test statistic	***p***-value	Effect size
**Demographic and anthropometric characteristics**							
**Age (years)**		*M* (*SD*)	24.80 (9.23)	23.28 (2.52)	*U* = 428	.281	*r* = .14
**Relationship status**					***χ***^***2***^ ***=*** 17.70***	**< .001**	*φ =* .53
In a relationship		*n* (%)	7 (20.0)	21 (72.4)	–	–	–
Not in a relationship		*n* (%)	28 (80.0)	8 (27.6)	–	–	–
**University entry qualification**					*χ*^*2*^ *=* 3.05	.081	*φ* ***=*** .22
Obtained		*n* (%)	29 (82.9)	28 (96.6)	–	–	–
Not obtained		*n* (%)	6 (17.1)	1 (3.4)	–	–	–
**Body mass index**		*M* (*SD*)	15.87 (2.85)	21.41 (2.19) ^a^	***U*** = 18***	**< .001**	*r* = .82
**Eating disorder-specific psychopathology**							
**Illness duration (years)**		*M* (*SD*)	9.03 (5,29) ^b^	–	–	–	–
**Comorbid disorders** ^**e**^							
n with ≥1 comorbidities		*n* (%)	14 (40.0)	–	–	–	–
MDD		*n* (%)	6 (17.14)	–	–	–	–
Social anxiety disorder		*n* (%)	4 (11.43)	–	–	–	–
Specific phobia		*n* (%)	3 (8.57)	–	–	–	–
Panic disorder with agoraphobia		*n* (%)	1 (2.86)	–	–	–	–
PTSD		*n* (%)	1 (2.86)	–	–	–	–
OCD		*n* (%)	3 (8.57)	–	–	–	–
BDD		*n* (%)	1 (2.86)	–	–	–	–
**EDE subscales**							
Restraint		*M* (*SD*)	3.17 (1.74)	.19 (.53) ^d^	***U*** = 46***	**< .001**	*r* = .79
Eating concern		*M* (*SD*)	2.49 (1.44)	.04 (.12) ^d^	***U*** = 19.5***	**< .001**	*r* = .86
Weight concern		*M* (*SD*)	2.45 (1.47)	.49 (.60) ^d^	***U*** = 87***	**< .001**	*r* = .73
Shape concern		*M* (*SD*)	3.14 (1.64) ^c^	.52 (.65) ^d^	***U*** = 65***	**< .001**	*r* = .69
**EDI-2 subscales**							
Drive for thinness		*M* (*SD*)	31.09 (8.56)	13.34 (6.13)	***U*** = 80***	**< .001**	*r* = .72
Bulimia		*M* (*SD*)	14.8 (6.86)	10.62 (3.69)	***U*** = 322*	**.012**	*r* = .31
Body dissatisfaction		*M* (*SD*)	38.74 (8,72)	26.21 (10.09)	***U*** = 172.5***	**< .001**	*r* = .57
**General Psychopathology**							
**BDI-II**	Total score	*M* (*SD*)	23.60 (10.16)	5.24 (4.93)	***t*** = −9.44***	**< .001**	*d* = 2.23
**STAI**	Trait-Anxiety	*M* (*SD*)	56.15 (9.45) ^c^	35.79 (9.51)	***t*** = −8.49***	**< .001**	*d* = 2.18

*M* Mean, *SD* Standard Deviation, *AN* Anorexia nervosa, *HC* Healthy control, *MDD* Major depressive disorder, *PTSD* Posttraumatic stress disorder, *OCD* Obsessive-compulsive disorder, *BDD* Body dysmorphic disorder, *EDE* Eating Disorder Examination, *EDI-2* Eating Disorder Inventory-2, *BDI-II* Beck Depression Inventory-II, *STAI* State-Trait Anxiety Inventory. ^a^*n* = 28. ^b^*n* = 32. ^c^*n* = 34. ^d^*n* = 26. ^e^ Multiple entries per person possible.

* *p* < .05; *** *p* < .001

### Associations of implicit drive for thinness with ED-specific and general psychopathology in participants with AN

Within the AN group, medium positive correlations emerged between implicit drive for thinness (pIAT D-scores) and EDE eating concern and EDE shape concern. In addition, the analyses yielded significant medium to large positive correlations between implicit drive for thinness (qIAT D-scores) and ED-specific psychopathology measures, i.e., all four EDE subscales as well as EDI-2 drive for thinness and EDI-2 body dissatisfaction (Table 2). No significant correlations were found between D-scores of qIAT or pIAT and general psychopathological measures.

**Table 2:** Associations of eating disorder-specific and general psychopathology with implicit drive for thinness in AN

D-score	BMI	EDE Restraint	EDE Eating concern	EDE Weight concern	EDE Shape concern	EDI-2 Drive for thinness	EDI-2 Bulimia	EDI-2 Body dissatisfaction	BDI-II Total score	STAI Trait anxiety
**pIAT**	.178 (*p* = .305)	.299 (*p* = .081)	.407* (*p* **= .015)**	.231 (*p* = .181)	.370* (*p* **= .031)**	.244 (*p* = .158)	−.035 (*p* = .844)	.241 (*p* = .163)	.146 (*p* = .401)	.096 (*p* = .588)
**qIAT**	−.136 (*p* = .436)	.379* (*p* **= .025)**	.371* (*p* **= .028)**	.371* (*p* **= .028)**	.359* (*p* **= .037)**	.437** (*p* **= .009)**	−.129 (*p* = .459)	.543** (*p* **= .001)**	.096 (*p* = .583)	.080 (*p* = .654)

*AN* Anorexia nervosa, *pIAT* Picture-based implicit association test, *qIAT* Questionnaire-based implicit association test, *BMI* Body mass index, *EDE* Eating Disorder Examination, *EDI-2* Eating Disorder Inventory-2, *BDI-II* Beck Depression Inventory-2, *STAI* State-Trait Anxiety Inventory. * *p* < .05; ** *p* < .01

### Group differences in implicit drive for thinness

#### Picture-based implicit association test

**Fig. 1:**
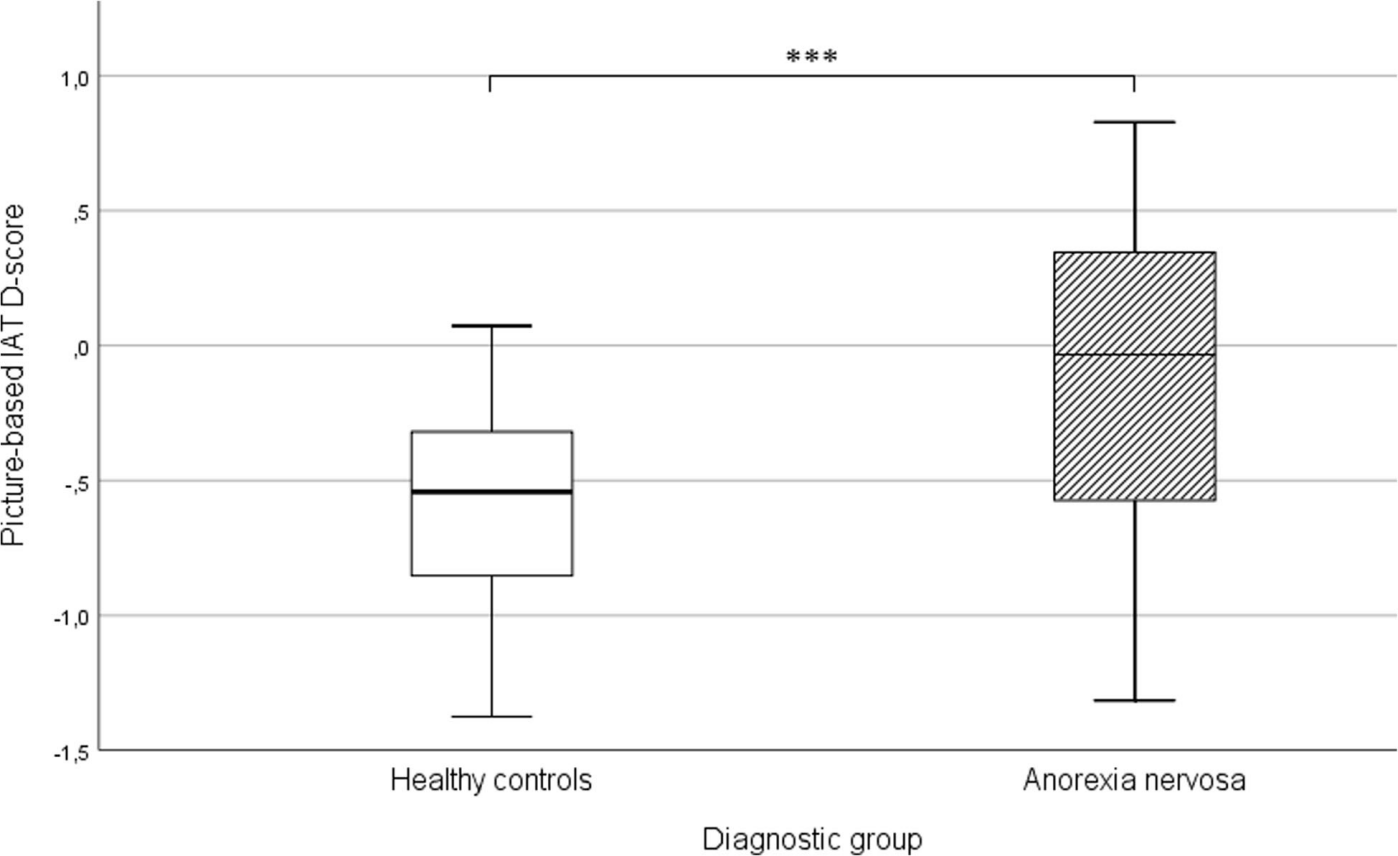
Picture-based implicit association test D-scores for HC and AN

There was a significant group difference in pIAT D-scores between participants with AN (*M* = −.13, *SD* = .60) and HCs (*M* = −.61, *SD* = .40), *t*(59.38) = − 3.87; *p* < .001; *d* = .94 (Fig. 1). Specifically, HCs showed a stronger association between *underweight* models and *negative* words compared to participants with AN. However, contrary to our first hypothesis, participants with AN did not exhibit positive biases toward *underweight* models; rather, the association was close to zero.

#### Questionnaire-based implicit association test

**Fig. 2:**
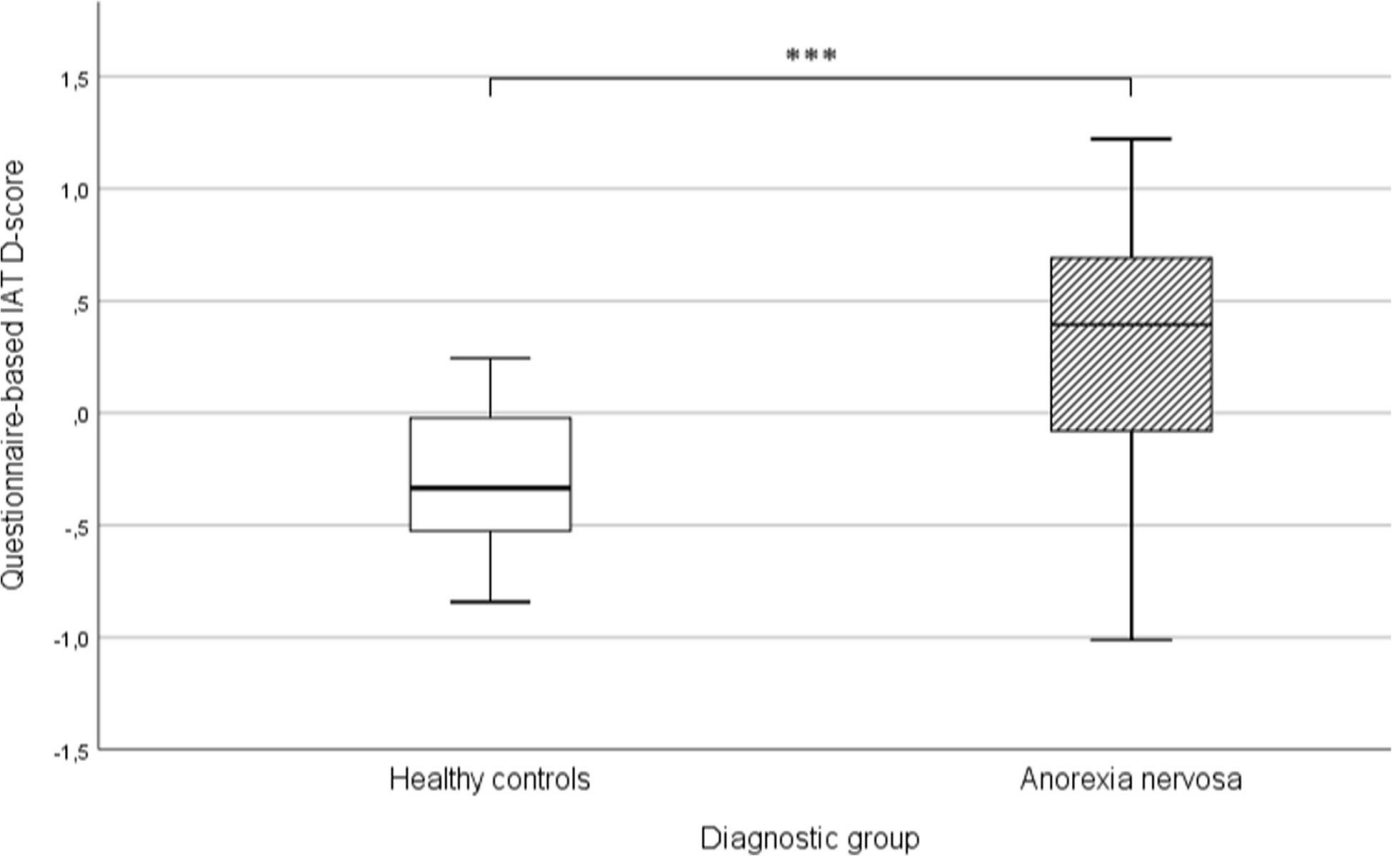
Questionnaire-based implicit association test D-scores for HC and AN

A significant group difference in the qIAT D-score between participants with AN (*M* = .28, *SD* = .54) and HCs (*M* = −.29, *SD* = .33) emerged, *t*(57.19) = − 5.15; *p* < .001; *d* = 1.24 (Fig. 2). Specifically, the AN group exhibited a strong association between *pro-dieting* statements and *true* statements, whereas HCs exhibited a strong association between *pro-dieting* statements and *false* statements.

## Discussion

This study examined associations of implicit measures of drive for thinness with explicit general and ED-specific psychopathological measures such as explicit drive for thinness in a German sample of participants with AN. Additionally, we examined differences in implicit attitudes toward thinness and dieting measured by the qIAT and pIAT between participants with AN and HCs, therewith partially replicating the US-American study by Izquierdo et al. [33] as a proof of concept in a German-language sample.

Partly confirming our first hypothesis, the results of the correlation analyses within the AN group revealed that higher qIAT D-scores were associated with more pronounced ED-specific psychopathology. PIAT D-scores showed no significant associations with most ED-specific psychopathological measures except the EDE eating concern and EDE shape concern scores. The results of both IATs showed significantly greater implicit drive for thinness among participants with AN compared to HCs, confirming our second hypothesis and indicating that IATs can reliably assess implicit drive for thinness. Specifically, in the qIAT, the AN group revealed a stronger positive bias toward *pro-dieting* statements, while pIAT results indicated neither positive nor negative biases toward *underweight* models in the AN group, with their actual D-score value being close to zero.

We found significant correlations of qIAT D-scores with explicit drive for thinness and body dissatisfaction as well as with the EDE subscale scores across all participants with AN. While viewing the qIAT D-scores as a proxy measurement of implicit FP, these results would be consistent with previous research indicating a less severe ED-specific psychopathology in individuals with NFP-AN vs. FP-AN [10, 11, 14, 15]. This also supports the approach that implicit FP represents a dimensional variable comparable to explicitly measured ED-specific psychopathology [36, 37]. The correlation of qIAT D-scores with the EDI-2 subscales drive for thinness and body dissatisfaction may additionally be explained by the proximity of the qIAT to those explicit measures due to relatedness of item content [33, 51], which might also be partly the case for EDE subscales restraint, eating, weight and shape concern [12]. The lack of correlations between the pIAT and most explicit ED-specific psychopathological measures could be explained by different types of indicators for the measured target construct [51]. Semantically complex stimuli, such as those in the qIAT, activate a wider range of self-referential psychological constructs, which then more closely correspond to the assessment in self-reports [51]. On a related note, while statements in the qIAT made it possible to map the thematic range of the four EDE subscales, the pIAT primarily addressed the shape concern subscale. Based on pictures, a viewer can only directly infer the figure of the person shown (not his or her weight), which may have boosted comparison processes in participants with greater degrees of FP. The correlative result regarding the eating concern subscale, however, is counterintuitive. Overall, however, these results are in line with the categorical findings by Izquierdo et al. [33].

Consistent with previous research [52], HCs showed positive associations between *normal-weight* models and *positive words* when confronted with underweight models in the pIAT, which could be interpreted as a healthy concept of body image ideal. In line with this, Izquierdo et al. [33] found a strong negative association between *underweight* models and *positive* words in HCs. In contrast, research regarding implicit biases in the AN group is heterogenous. While Ahern et al. [32] found that women with a stronger drive for thinness had positive implicit biases toward *underweight* models, Izquierdo et al. [33] observed a negative association toward *underweight* models in the AN group. Results of the present study showed a pIAT D-score value close to zero in the AN group, which can be interpreted as a lack of association [44]. This may be explained by an identification bias, insofar as women with AN display greater self-deprecating double standards in body fat rating than women without an ED [53]. Consequently, their perception may be distorted only when looking at their own bodies and not when looking at other bodies like in the pIAT. Izquierdo et al. [33] suggested that participants’ socially established ideals of thinness do not correspond with the images of underweight models used in the pIAT, which might have led to less relevance for one’s own body image and associated implicit biases. This is further supported by findings on implicit associations toward emaciation, suggesting that individuals with AN might display complex implicit biases toward thinness [33, 54]. In order to investigate this approach, future pIATs should consider inlcuding pictures of participants’ own vs. others’ bodies—possibly in varying levels of thinness.

Average qIAT D-scores in HCs also match previous research [33] indicating an association between *pro-dieting* and *false* statements. This is in line with the above-mentioned healthy approach toward *underweight* and *normal-weight* models in HCs. Consistent with previous research [31], the AN group presented a strong positive implicit bias toward *pro-dieting* statements in the qIAT. Compared to the pIAT results, the qIAT with its self-referential statements might have led to a greater relevance for their own body images and implicit beliefs. In addition, Friedman et al. [51] support that the qIAT is a more appropriate measure of attitudes about the self—with better convergent validity with explicit measures—compared to other implicit measures. Due to the increased self-relevance and proximity to actual attitudes, the qIAT seems to provide more information regarding the implicit measurement of drive for thinness [33].

In light of the categorical findings by Izquierdo et al. [33] that failed to reveal differences in implicit drive for thinness between individuals with AN reporting and not reporting FP, a significant relationship between implicit and explicit drive for thinness and other ED-specific measures seems contradictory. These inconsistencies may be attributable to changes in reasons for food intake restriction during the course of AN [55], besides fear of weight gain, e.g., need for control [56, 57]. As a consequence, we might have diverse samples of individuals with varying degrees of FP that can be better represented by dimensional metrics. Additionally, the consideration of FP and drive for thinness as one end of a single dimension, using the latter as sort of a proxy for the former, might also have contributed to the discussed inconsistencies. A recent study by Rodgers, DuBois, Frumkin, and Robinaugh [58] has shown that the frequently interchangeably measured concepts FP and drive for thinness seem to be distinct constructs and should not be considered as a single symptom. Rodgers and colleagues [58] further highlight that these constructs also have distinct association patterns within the network of ED symptoms. This assumption would lead to a high relevance of assessing those constructs separately in future studies, and, therewith, perhaps allow for categorical data to match our dimensional data.

Some limitations of this study must be acknowledged. Since the analyses of the current study have been performed in small samples, the generalizability of results to the AN population is limited. Moreover, 14 participants with AN had at least one comorbid mental disorder, and the impact of comorbidity on the results cannot be quantified. However, given that comorbidity is the rule rather than the exception in AN, the recruitment procedure provided a representative group of the AN population. Furthermore, the IAT is susceptible to contextual influences known from explicit self-reports [23], although these are considerably reduced due to the improved evaluation algorithm on IATs and corresponding recommendations regarding the analysis [44]. The strengths of this study are the thorough diagnosis of all participants and the standardized application of IATs, allowing for a partial replication and substantial extension of the work by Izquierdo et al. [33].

## Conclusion

The present study is one of few investigations of FP in AN with a dimensional approach based on an implicit task. Moreover, the present study partially replicated a study by Izquierdo et al. [33] which compared implicit associations between individuals with low-weight EDs. In regard to this categorical approach, the present findings are consistent with previous research. Thus, this study represents a proof-of-concept study for the implicit measurement of a construct that might serve to further tailor treatment strategies to individual needs based on the level of FP or drive for thinness. Future studies should employ the paradigm in the German version to assess samples of individuals with different levels of FP as has been done in an American sample [33]. Since in our sample only seven individuals could have been assigned to the subgroup NFP-AN, we refrained from this analysis.

Furthermore, our study underscores the importance of using a dimensional perspective on relevant ED-specific constructs, such as drive for thinness and FP in AN. Even if these constructs cannot be considered as congruent, they are closely related via superordinate concepts such as body dissatisfaction or negative body image. As part of this, the body functions as a self-value determining construct leading to food intake restriction. Therefore, intensive research on drive for thinness and FP as well as on its dimensional analyses remains of great importance. Future studies could focus on longitudinal analyses in AN using implicit measures to effectively monitor implicit manifestations and courses. If there are samples of individuals with lower degrees of or non-existent FP, individually adapted treatment approaches such as the exploration and cognitive modification of alternative rationales for food intake restriction might be more promising. As a therapist, addressing unreported FP and taking these statements seriously could improve the therapeutic relationship as well as the patients’ compliance, which in turn could also have a positive impact on increased drop-out rates and low insights into their condition that was previously found in individuals with NFP-AN [11]. For individuals lacking insight into their underlying FP, a treatment focused on the detection and regulation of emotions could increase treatment efficacy. Considering that a discrepancy between explicit and implicit attitudes can lead to a detrimental effect on change and intention to change, interventions should aim for a concordance of explicit and implicit attitudes. In individuals who lack conscious awareness of or deny underlying FP, implicit measurements could shed light on it and consequently support treatments to improve perception and communication of FP.

## Data Availability

The local ethics committee stipulated that data must not be passed on to third parties. Therefore, data sharing is not applicable to this article.

## Abbreviations

AN: Anorexia nervosa
ANOVA: Analysis of variance
ARFID: Avoidant/restrictive food intake disorder
BDI-II: Beck Depression Inventory-II
BMI: Body mass index
DSM-IV/ -5: Diagnostic and Statistical Manual of Mental Disorders fourth edition/ fifth edition
ED: Eating disorder
EDE: Eating Disorder Examination
EDI-2: Eating Disorder Inventory second edition
FP: Fat phobia; Fear of weight gain
HC: Healthy Control
IAT: Implicit association test
NFP: Non-fat phobia; No fear of weight gain
pIAT: picture-based implicit association test
qIAT: questionnaire-based implicit association test
SCID: Structured Clinical Interview for DSM-IV
STAI(−T): State-Trait Anxiety Inventory (trait scale)
